# FFPE samples from cavitational ultrasonic surgical aspirates are suitable for RNA profiling of gliomas

**DOI:** 10.1371/journal.pone.0255168

**Published:** 2021-07-22

**Authors:** Cristina Alenda, Estefanía Rojas, Luis M. Valor

**Affiliations:** 1 Instituto de Investigación Sanitaria y Biomédica de Alicante (ISABIAL), Alicante, Spain; 2 Departamento de Patología, Hospital General Universitario de Alicante, Alicante, Spain; 3 Laboratorio de Apoyo a la Investigación, Hospital General Universitario de Alicante, Alicante, Spain; Universita degli Studi di Torino, ITALY

## Abstract

During surgical procedures for gliomas, tissue material obtained from cavitational ultrasonic surgical aspirators (CUSAs) is generally discarded but can actually exceed the amount and quality of certain tumour core resections (TCRs). Despite reports indicating the suitability of CUSA-derived material for diagnosis and research, its use is still marginal. We extended these conclusions to formalin-fixed, paraffin-embedded (FFPE) samples, the most common format for archival tumour tissue in anatomical pathology departments, by conducting for the first time RNA-seq analysis in CUSA aspirates. We compared the molecular diagnosis of somatic mutations used in the clinical routine and the gene expression profiles of fixed solid material from CUSA aspirates and TCRs from the same patients in selected gliomas encompassing grades II to IV. Despite the characteristic heterogeneity of gliomas, we found substantial similarities between the corresponding aspirates and TCRs that included transcriptional signatures associated with glioma subtypes. Based on these results, we confirmed that CUSA-fixed biomaterials from glioma surgeries are appropriate for downstream applications and biomarkers screening.

## Introduction

After diagnosis, surplus tissues from glioma surgical resections are used for subsequent molecular, biochemical and cellular studies. However, the small size of the excised mass and extensive necrosis or vascularization can limit the amount and integrity of the material for research activities. The cavitational ultrasonic surgical aspirator (CUSA) is currently indispensable in neurosurgery departments to partially fragment the tumour before surgical resection of the intracranial mass defined by magnetic resonance imaging. The action of CUSA minimizes the damage of surrounding brain tissue and blood vessels, increasing the safety and surgical precision while reducing the duration of the brain surgery and the probability of postoperative complications [1–3].

These fine fragments are aspirated together with the irrigation liquid and discarded after the operation as they are generally regarded as biological waste. However, early reports discovered the suitability for diagnosis of the solid material obtained by the CUSA from diverse brain tumours based on both cytomorphology and immunohistochemistry studies [4, 5], and recommendations for the use of CUSA-derived material for histopathological inspection has been proposed [6]. In glioblastoma (GB), this type of material reproduces the heterogeneity of the tumour core resections (TCR), shares similar genetic and transcriptional alterations, and contains viable cells for the establishment of primary cultures of glioma stem cells [7–9]. This is of interest, as the volume of CUSA-derived tissue is usually larger than that of the TCR, increasing the sensitivity of diagnosis and the availability of material for downstream research. More recently, surgical aspirates have been found to be able to provide extracellular vesicles directly from the brain, otherwise inaccessible, as a potential source of biomarkers in glioma [10, 11]. To the best of our knowledge, previous studies did not examine these aspirates by RNA-seq, the current gold-standard of transcriptomics profiling, from formalin-fixed, paraffin-embedded (FFPE) samples that can be easily archived by anatomical pathology departments from a variety of glioma subtypes. In the present study, we conducted a pilot study to establish proof of principle with selected examples about the suitability of using FFPE-solid material from the CUSA for the diagnosis, biomarker discovery and research of gliomas, including advanced transcriptomics studies.

## Materials and methods

### Ethics approval and patients

This study was approved by the local ethics committee (Comité de Ética de Investigación Clínica con Medicamentos del Hospital General Universitario de Alicante) according to the ethical principles of the Declaration of Helsinki and according to national and regional law regulations concerning biomedical research with human samples, personal data protection and the use of biobank services. All participants gave written informed consent.

**Fig 1:**
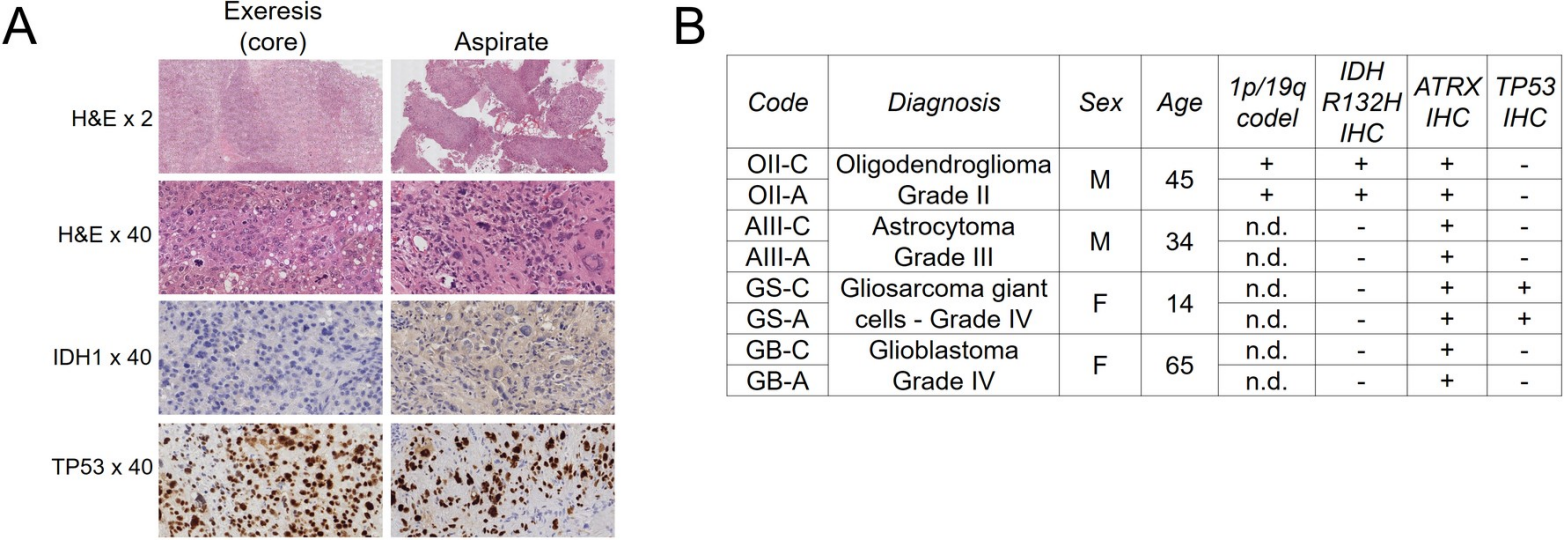
The diagnosis of glioma aspirates is highly similar to their corresponding core resections. *A*, Representative immunohistochemistry images of the gliosarcoma of giant cells used in this study. H&E, haemotoxylin and eosin. *B*, Diagnosis of the samples used in the present study. C = tumour core resection. A = cavitational ultrasonic surgical aspirate. Age = Age at diagnosis.

Biomaterial was obtained after surgical procedures that included ultrasound aspiration using a CUSA Curved Extended Standard 36 KHz tip with CUSA Excel+ equipment (Integra LifeSciences). These aspirates were filtered using a strainer to retain the solid tissue that was subsequently fixed with formalin and embedded in paraffin. The main characteristics of the patients (age, sex, histological and molecular diagnosis) are shown in Fig 1. Samples and data from patients included in this study were provided by the BioBank ISABIAL, integrated in the Spanish National Biobanks Network and in the Valencian Biobanking Network. They were processed following standard operating procedures with the appropriate approval of the Ethical and Scientific Committees.

### Immunohistochemistry

Histological and molecular diagnoses were performed following the WHO 2016 classification of CNS tumours [12]. Immunohistochemical staining was performed using a Dako Omnis System (Dako, Agilent Technologies) according to the manufacturer’s instructions. Antigen retrieval was performed using EnVision FLEX Target Retrieval Solution, High pH (Dako, Agilent Technologies). Sections were incubated for 25 minutes with primary antibodies against ATRX (clone AX-1, Dianova1/300), IDH1 R132H (clone H09, Dianova 1/20) and TP53 (clone DO-7, Dako). After chromogenic visualization using EnVision FLEX/HRP (Dako, Agilent Technologies), the slides were counterstained with haematoxylin.

### RNA extraction and RNA-seq

We deparaffinised 3 slices of 10 μm thickness from each FFPE block according to the xylene protocol described in the RNeasy FFPE kit (Qiagen) handbook. Total RNA was obtained after purification and DNase digestion using the RNeasy FFPE kit (Qiagen) following the manufacturer’s instructions, and was sent to an external sequencing service (Unidad de Genómica, Cabimer). The starting amount of total RNA was 100 ng (measured using the Qubit RNA HS Assay (ThermoFisher)) following Illumina’s recommendations for preparing the sequencing library using the TruSeq RNA Exome library kit (Illumina), which resulting fragments were measured in a Bioanalyzer DNA High Sensitive chip (Agilent). Deep sequencing was performed on a NextSeq 500 (Illumina) in a configuration of paired-end and 75 bp-length reads. Reads were aligned to the human genome (hg19 build) using TopHat Alignment software (v1.0.1 in BaseSpace), resulting in the percentage of aligned reads and number of genes with aligned reads shown in Table 1. MultiQC in DRAGEN RNA pipeline v3.7.5 (Illumina) was used to calculate the GC content (in %) across the reads.

**Table 1:** Characteristics of the samples used in the present study.

*Code*	*Diagnosis*	*RIN*	*DV200*	*Reads*	*% aligned*	*Genes with reads*
OII-C	Oligodendroglioma, IDH-mutant, 1p/19q-codeleted Grade II	2.30	44	40,109,782	90.44	33,371
OII-A		2.40	49	41,087,894	87.68	30,906
AIII-C	Astrocytoma, IDH-wildtype Grade III	2.40	32	37,484,635	86.32	32,638
AIII-A		2.40	28	45,693,652	86.31	32,639
GS-C	Gliosarcoma, IDH-wildtype Grade IV	2.00	53	44,422,490	91.43	30,970
GS-A		1.90	71	40,142,882	92.84	32,394
GB-C	Glioblastoma, IDH-wildtype Grade IV	2.30	38	39,110,142	89.14	29,967
GB-A		2.10	43	39,923,403	86.57	31,281

C = tumour core resection (TCR); A = cavitational ultrasonic surgical aspirate; RIN = RNA integrity number; DV200 = percentage of fragments >200 nt; Reads = numbers of paired reads; % aligned = % aligned reads to genome of reference.

### Additional bioinformatics

The following Bioconductor-based software packages were used for conversion of BAM files (*RSamtools*), gene annotation (*GenomicFeatures* and *GenomicAlignments* using the GTF file Homo_sapiens.GRCh37.87.chr.gtf extracted from the Ensembl website, https://www.ensembl.org/) and GC content-based normalization of the data (*EDASeq* [13], “median” method). This normalization was conducted for all the samples after the removal of genes with null or very low numbers of reads (<10 across all samples). Additionally, we only considered genes with consensus information regarding gene length and GC content. We obtained a final number of 26,309 transcripts (52% of the total annotated transcripts in the gtf file) for subsequent analyses. Seqmonk software was used for exon mapping of the reads (https://www.bioinformatics.babraham.ac.uk/projects/seqmonk/). Clustering of the data was conducted using the *rgl* package (http://cran.r-project.org/package=rgl) for 3-D principal component analysis (PCA).

To inspect for proneural and mesenchymal transcriptional signatures previously described in gliomas in the gene profiles derived from the FFPE tissues, we used the common genes obtained from “TCGA_unified_CORE_ClaNC840.txt” file (https://gdc.cancer.gov/about-data/publications/gbm_exp, [14]) and Supplementary Table S1 of ref. [15]. Thus, we considered as proneural genes *ASCL1*, *DLL3*, *EPHB1*, *FXYD6*, *GABRA3*, *GRIA2*, *KIF21B*, *KLRC3*, *MAPT*, *OLIG2*, *P2RX7*, *SCG3*, *SEZ6L*, *SLC1A1*, *SORCS3* and *TTYH1*, and as mesenchymal genes *EFEMP2*, *EHD2*, *EMP3*, *FCGR2A*, *FES*, *HK3*, *ITGA5*, *MVP*, *NRP1*, *PLAU*, *PLAUR*, *RRAS*, *S100A11*, *SERPINA1*, *SERPINE1*, *SLC16A3*, *THBD* and *TIMP1*.

Transcriptomics differential expression was determined by using DESeq2 software [16] in two types of comparisons: (i) between grade IV and grade II/III samples for each type of tissue (TCR or aspirate) (n = 2 for each condition), and (ii) between TCR and CUSA-derived material, independent of the diagnosis and degree (n = 4 for each condition). In the first comparison (IV *vs* lower grade), we filtered those differentially expressed genes (DEGs) belonging to X and Y chromosome genes, as grade IV tumours and lower grade gliomas were obtained from women and men, respectively. Then, the resulting DEGs were contrasted with those retrieved from external data: (i) RNA-seq data from The Cancer Gene Atlas (TCGA) consortium as deposited in the Genomic Data Commons (GDC) website (https://portalgdc.cancer.gov) and analysed according to the pipeline described previously using “TCGAbiolinks” software [17] (n = 166 for grade IV and n = 528 for gliomas of grades II and III); and (ii) gene expression microarray data (Human Genome U133 Plus 2.0 platform, Affymetrix) from the REMBRANDT study [18] as deposited in GeneBank Accession number GSE108474 and analysed using the Bioconductor *affy* [19] and *limma* [20] packages (n = 218 for grade IV and n = 216 for gliomas of grades II and III). In the second comparison (aspirates *vs* TCR), we explored the gene expression patterns of the DEGs across anatomical structures of GB tumours contained in the RNA-seq data of the Ivy Glioblastoma Atlas Project (Ivy GAP, https://glioblastoma.alleninstitute.org/) [21]. To avoid bias due to different degrees of expression between genes, we used the Z-score values from the website that estimated the variation from the mean values for each gene.

## Results

From our FFPE-tissue collection, we selected four types of gliomas for which both solid material of TCR and CUSA were available from the same patients: an oligodendroglioma, IDH-mutant and 1p/19q-codeleted (OII), an astrocytoma, IDH-wildtype (AIII), and two GBs, IDH-wildtype; to distinguish these GBs throughout this work, one of them will be referred hereafter as GS (for gliosarcoma). We confirmed that the routine diagnosis of TCR and corresponding CUSA samples were the same (Fig 1), indicating that the CUSA biomaterial can reproduce the profiles of clinically relevant somatic mutations of TCR.

**Fig 2:**
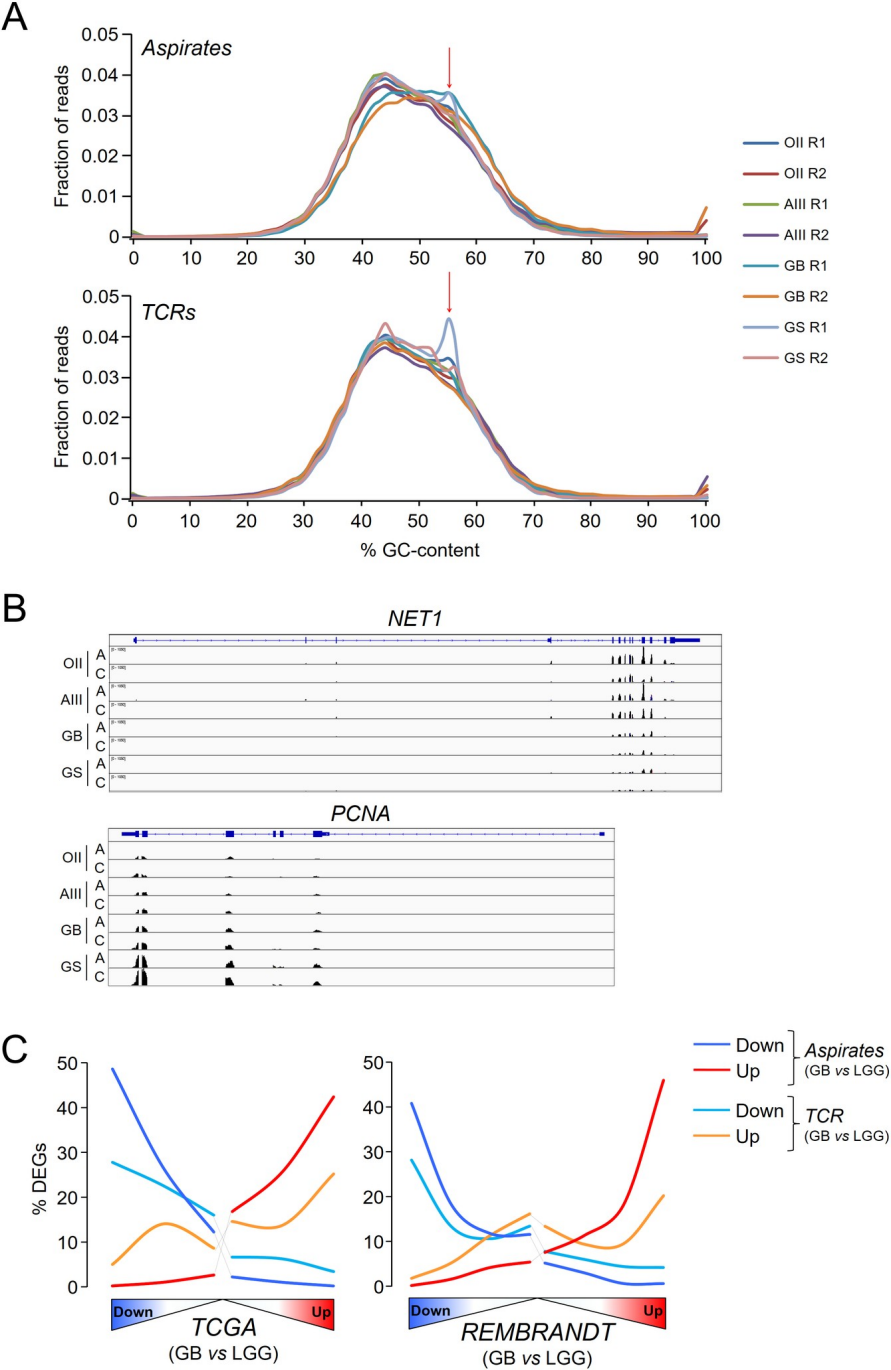
Transcriptomics analysis can be performed using FFPE glioma samples. *A*, Distribution of GC-content across reads in the FFPE samples used in the study. Because of the paired-end sequencing, each sample produced two fastq files, R1 and R2. The red arrow indicates a secondary aberrant peak. *B*, Representative RNA-seq tracks for a downregulated gene (*NET1*, *neuroepithelial cell transforming gene 1*) and an upregulated gene (*PCNA*, *proliferating cell nuclear antigen*) in grade IV tumours related to lower grade gliomas (LGG, II/III). C = tumour core resection. A = cavitational ultrasonic surgical aspirate. *B*, In the upper panels, percentages of DEGs between grade IV and lower-grade gliomas in the FFPE samples across the whole GB transcriptomes from the TCGA and REMBRANDT cohorts. These transcriptomes were ranked according to the significance and direction of the gene expression change compared to lower-grade gliomas and divided into bins of 2000 genes. The number of DEGs in each bin was then counted and referred to as a % of the total number of DEGs. All distributions were significantly different from random distribution of DEGs (χ^2^, *P*< 0.00001).

To further determine the degree of similarities between the TCR and CUSA-derived solid material, we undertook a genome-wide survey of the gene expression profiles. After deparaffinization and RNA purification, we noticed that its integrity was expectedly compromised (RNA integrity number (RIN) ~2.2, percentage of fragments >200 nt (DV200) ~45, see Table 1 for details) compared to fresh-frozen tissue, typically RIN ~7 and DV200 ~85. However, RNA degradation did not impede the preparation of appropriate libraries for next-generation sequencing, as evidenced by the consistent size of the resulting fragments (260–280 bp) and the total number of sequenced reads (Table 1) that mainly mapped into exons (91–94% of the reads). Contrary to previous studies of RNA-seq in FFPE samples in which abnormal peaks of GC-content surpassed the peak corresponding to the expected distribution of 40–60% of GC-content across the reads [22, 23], all our samples exhibited a prominent peak around 40–55% of GC-content (Fig 2A). Only three samples showed an abnormal peak around 56% of GC-content that has been attributed to read mapping to intronic regions [23], indicating a reasonable good mapping into exons as already mentioned above (Fig 2B). After gene annotation and filtering (see Materials and Methods), we estimated the percentage of expressing coding and non-coding RNAs based upon the Ensembl “gene biotype” classification in TCR and CUSA-derived samples: approximately two thirds of the transcripts were from protein coding genes in both types of samples (Table 2).

**Table 2:** Percentages of transcript types detected in tumour core resections (C) and cavitational ultrasonic surgical aspirates (A).

*RNA type*	*C*	*A*
Protein-coding	63.6	64.4
Pseudogenes	17.7	16.9
Long non-coding	11.3	11.3
Small RNAs	2.4	2.3

**Fig 3:**
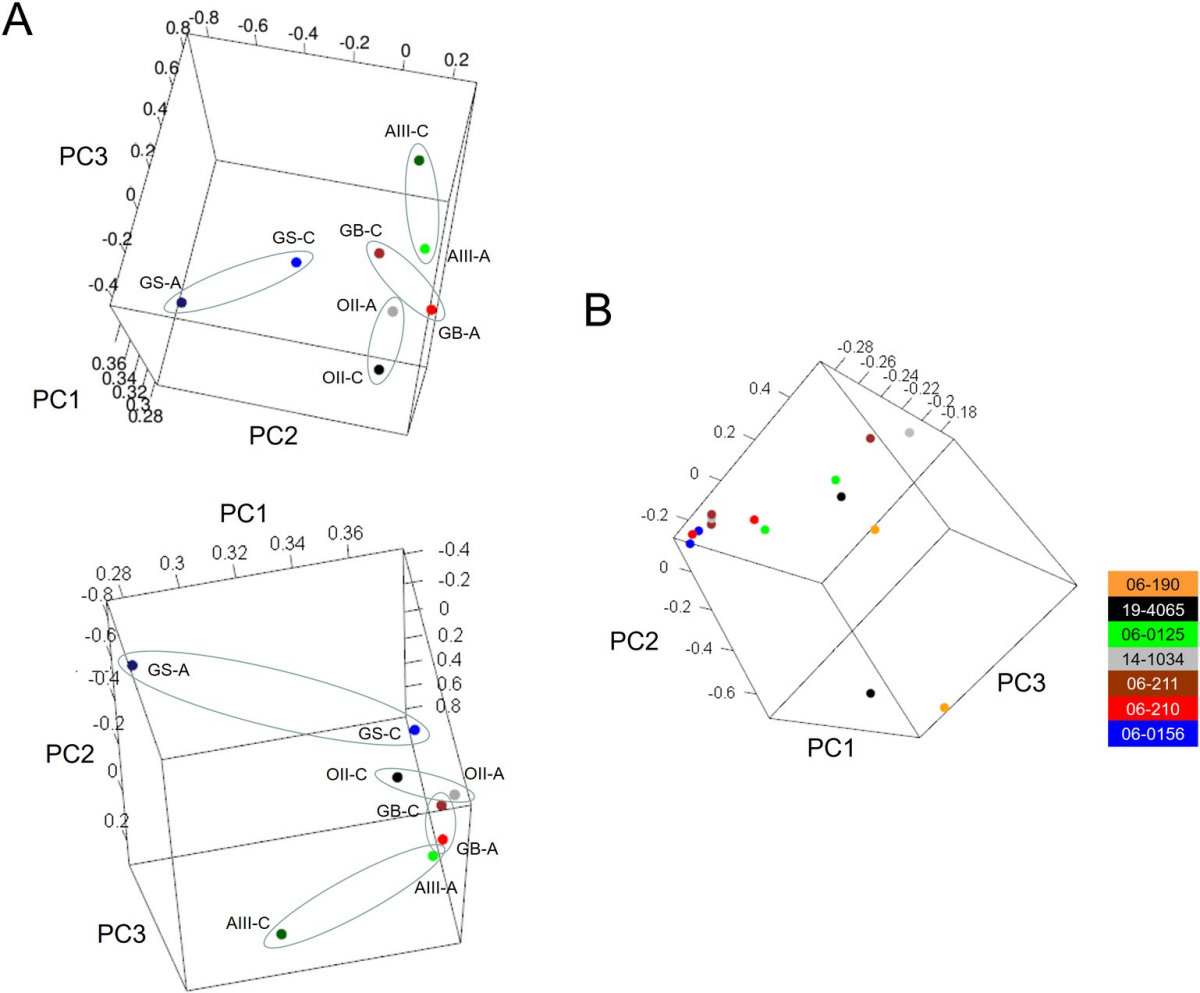
The transcriptional profiles of the tumour aspirates and core resections are similar. 3-D PCA of our samples (*A*) and the samples from the TCGA repository (*B*). C = tumour core resection. A = cavitational ultrasonic surgical aspirate. The colouring scheme indicates the patient ID of the TCGA from which the different tumoural resections were obtained.

To verify that the sequencing data in the FFPE material reproduced the transcriptional profiles of fresh gliomas, we compared the differential expression between GB tumours and lower grade gliomas (II and III) from TCRs and aspirates separately, and from external datasets derived from fresh tissue (TCGA and REMBRANDT cohorts). To avoid any bias due to different number of DEGs after applying thresholds of fold change and adjusted *P*-values, we selected the top 500 DEGs of each pair-wise comparison for further analysis (all these genes had at least unadjusted *P*-values < 0.05). Noticeably, the expression patterns from aspirate samples were highly similar to those obtained using the datasets from TCGA and the REMBRANDT cohorts after performing the same type of comparison: 60–70% of the down- and upregulated genes retrieved in our samples were also differentially expressed in the same direction of change in these public cohorts, indicating that FFPE aspirated tissues reasonably reproduced the full transcriptomes of fresh tissues, and allowed for the application of advanced analytical approaches in glioma studies (Fig 2C). The PCA of the sequencing data determined that, in general, the profiles of the aspirates were close to the profiles of their corresponding TCRs (Fig 3A), although the GS showed the highest divergence between the aspirates and TCR, probably due to the mixture of glial and sarcomatous components that is characteristic of this GB variant. Similar degrees of variability were also found in different resections from the same patients collected in the TCGA database (Fig 3B), confirming that differences in the transcriptional profiles of our samples accounted for the expected heterogeneity of gliomas.

**Fig 4:**
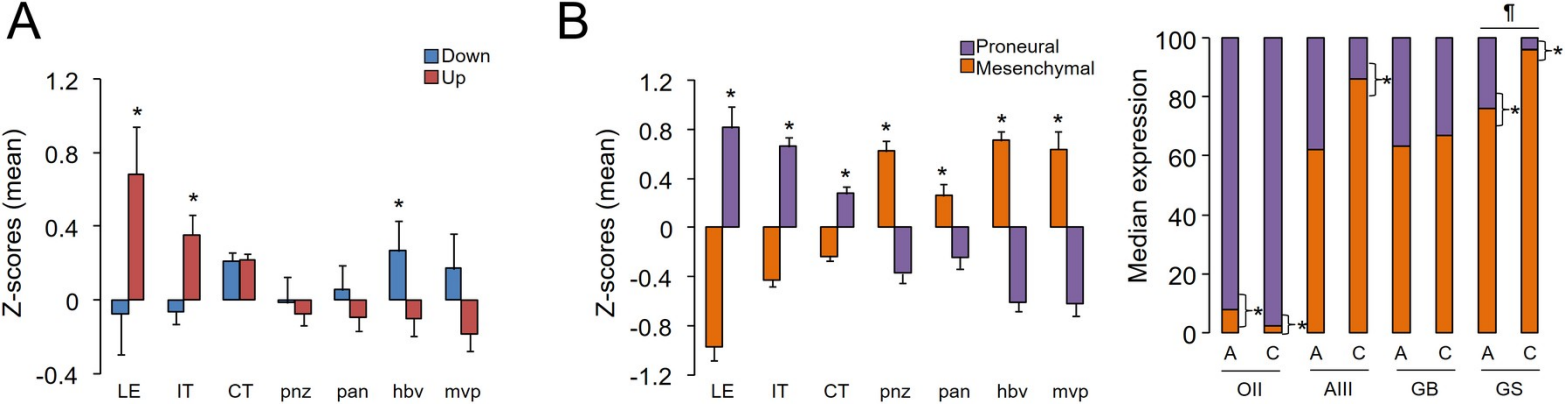
Tumour aspirates and core resections show slight regional differences. *A*, Mean ± s.e.m. of the Z-scores obtained for the DEGs across the tumoural regions defined in the Ivy GAP website: LE, leading edge; IT, infiltrating tumour; CT, cellular tumour; pnz, perinecrotic zone; pan, pseudopalisading cells around necrosis; hbv, hyperplastic blood vessels; mvp, microvascular proliferation (see ref. [21] for further explanations). *, *P*< 0.05, Mann-Whitney’s test between the mean values of down- and upregulated genes within each compartment. *B*, On the left, the same analysis was performed with the proneural and mesenchymal markers defined in Materials and Methods; on the right, the median normalized expression values of these markers in our FFPE samples. *, *P*< 0.05, Mann-Whitney’s test between the overall expression values of proneural and mesenchymal genes within each sample. ¶, *P*< 0.05, Mann-Whitney’s test between the overall expression values of mesenchymal genes between the aspirate and the TCR.

Next, we searched for a consistent signature associated with CUSA-derived material independent of the glioma subtype by determining the differential expression between all aspirates and all TCR samples. Only 15 and 26 transcripts were down- and upregulated in aspirates related to the core resections, respectively (due to the low number of DEGs we considered an adjusted *P*-value <0.1). After this pair-wise comparison, we did not retrieve genes previously associated with GB, except for *SLC13A5*, which can show perturbed gene expression and methylation patterns in this cancer type [24]. Among the upregulated genes, we observed two tumour-specific antigen genes of the *GAGE12* family that can be relevant in the progression of gastric carcinoma [25]. Thanks to the Ivy GAP database [21], we examined whether there existed a regionalization pattern of these DEGs within glioma tumours. Interestingly, the downregulated genes in aspirates (*ARHGAP28*, *CKAP2*, *EDARADD*, *HOXD8*, *RORC*, *SLC5A1*, *ZNF486*, *ZNF92* and *LOC100132396* (*ZNF705B*)) were generally more expressed in vascularized zones such as hyperplastic blood vessels, whereas the upregulated genes (*ABCB9*, *ACY3*, *CEACAM5*, *GAGE12F*, *GAGE12G*, *GJC2*, *LOC642131* (*IGHV4OR15-8*), *NAT8L*, *PHYHD1*, *PI16*, *TACR3* and *SLC13A5*) tended to be more highly expressed in the peripheral areas of the tumours: the leading edge, as the outermost boundary of the tumour, and the infiltrating tumour, as the intermediate zone between the leading edge and the cellular tumour (Fig 4A), as defined in the original publication [21]. This regional profile was reminiscent of the behaviour observed for markers of proneural and mesenchymal gliomas (see Materials and Methods), in which the former genes were enriched in peripheral areas while the latter genes were more highly expressed in vascularized regions (Fig 4B left). Although none of these markers was found among the DEGs between aspirates and TCRs, we observed that at least in the case of AIII and GS, mesenchymal genes were significantly expressed at higher levels in TCRs than in their corresponding aspirate tissues, in agreement with their enrichment in internal regions of GB tumours (Fig 4B, right). Overall, these results suggested that the potential signature specifically associated with aspirate tissues across glioma subtypes might be related to the peripheral regions of the tumours as a result of the surface fragmentation of the solid tumour by the CUSA.

## Discussion

Despite cumulative evidence in favour of using fragmented material from ultrasound aspirates obtained in brain surgeries [4, 5, 7–9], its use in diagnosis and research is still scarce in the literature. In this study, we went a step further by demonstrating that the similarities between CUSA-derived samples and their corresponding TCRs can also be verified in FFPE tissues, the most extended format of tumour archiving in hospitals, allowing their revisitation years after collection. Current improvements in library preparation and deep sequencing enable gene expression exploration with sufficient guarantees in FFPE tissues, despite RNA degradation, cross-linking and chemical modifications during fixation and storage ([26] and references therein). Despite using a small number of samples, we were able to confirm that our samples can be analysed in a similar manner to regular TCRs; for example, we were able to retrieve gene expression changes in GBs compared to lower grade gliomas, such as *POLR2L* [27] and *ZMYND11* [28], as potential candidates for glioma malignancy, and to classify the samples into molecular glioma subtypes with differential clinical outcomes (Fig 4) according to the predominance of associated transcriptional signatures [14, 15].

In contrast, we failed to reproduce the gene expression differences reported between TCRs and CUSA-derived samples in a previous work that indicated an enrichment of tumourigenic-related genes and pathways in aspirates in a microarray analysis of fresh tissues [9], which can be contradictory to the regional localization of proneural and mesenchymal genes that we observed using the information of the Ivy GAP Database (Fig 4). This report was focused on GB samples that also included non-matching samples (n = 4 for TCR and n = 9 for aspirates) that may explain the extensive differential expression of >300 genes [9]. In our hands, we found an apparent over-representation of genes from the peripheral regions of the tumours in aspirates, which are characterized by a large proportion of normal cells [21] that is expected to express higher levels of proneural genes, whereas TCRs tended to contain more internal portions or tissue adjacent to vascularisation processes. In any case, we observed minimal gene expression differences between both origins of biomaterials in matched samples, suggesting that aspiration material can substitute tumour cores to study the most relevant aspects of the biology of gliomas. However, we should be cautious in our conclusions as we need a larger number of samples to determine a comprehensive and more reliable gene expression signature linked to tumour aspirates.

In conclusion, our study successfully evaluated the suitability of FFPE solid samples from surgical aspirates for routine diagnostic and transcriptomic analysis in different types of gliomas, without the limitations due to the lack of availability of either fresh or core material, to fuel the discovery of novel biomarkers with diagnostic and prognostic utility in gliomas.
